# Variation in Accessory Genes Within the *Klebsiella oxytoca* Species Complex Delineates Monophyletic Members and Simplifies Coherent Genotyping

**DOI:** 10.3389/fmicb.2021.692453

**Published:** 2021-07-02

**Authors:** Amar Cosic, Eva Leitner, Christian Petternel, Herbert Galler, Franz F. Reinthaler, Kathrin A. Herzog-Obereder, Elisabeth Tatscher, Sandra Raffl, Gebhard Feierl, Christoph Högenauer, Ellen L. Zechner, Sabine Kienesberger

**Affiliations:** ^1^Institute of Molecular Biosciences, University of Graz, Graz, Austria; ^2^BioTechMed-Graz, Graz, Austria; ^3^Diagnostic and Research Institute of Hygiene, Microbiology and Environmental Medicine, Medical University of Graz, Graz, Austria; ^4^Division of Gastroenterology and Hepatology, Department of Internal Medicine, Medical University of Graz, Graz, Austria; ^5^Field of Excellence BioHealth, University of Graz, Graz, Austria

**Keywords:** bacterial phylogeny, *Klebsiella oxytoca* species complex, taxonomic classification, necrotizing enterocolitis, bacterial cytotoxicity, intestinal disease

## Abstract

Members of the *Klebsiella oxytoca* species complex (KoSC) are emerging human pathogens causing infections of increasing significance especially in healthcare settings. KoSC strains are affiliated with distinct phylogroups based on genetic variation at the beta-lactamase gene (*bla*_OXY_) and it has been proposed that each major phylogroup represents a unique species. However, since the typing methods applied in clinical settings cannot differentiate every species within the complex, existing clinical, epidemiological and DNA sequence data is frequently misclassified. Here we systematically examined the phylogenetic relationship of KoSC strains to evaluate robustness of existing typing methods and to provide a simple typing strategy for KoSC members that cannot be differentiated biochemically. Initial analysis of a collection of *K. oxytoca*, *K. michiganensis, K. pasteurii*, and *K. grimontii* strains of environmental origin showed robust correlation of core phylogeny and blaOXY grouping. Moreover, we identified species-specific accessory gene loci for these strains. Extension of species correlation using database entries initially failed. However, assessment of average nucleotide identities (ANI) and phylogenetic validations showed that nearly one third of isolates in public databases have been misidentified. Reclassification resulted in a robust reference strain set for reliable species identification of new isolates or for retyping of strains previously analyzed by multi-locus sequence typing (MLST). Finally, we show convergence of ANI, core gene phylogeny, and accessory gene content for available KoSC genomes. We conclude that also the monophyletic members *K. oxytoca*, *K. michiganensis*, *K. pasteurii* and *K. grimontii* can be simply differentiated by a PCR strategy targeting *bla*_OXY_ and accessory genes defined here.

## Introduction

The genus *Klebsiella* belongs to the family of Enterobacteriaceae and comprises multiple species. *Klebsiella pneumoniae* and species of the *Klebsiella oxytoca* complex are currently responsible for most human illnesses. Accurate identification of these pathogens is thus important for diagnosis, treatment, and epidemiological surveillance of infections. The *K. oxytoca* species complex (KoSC) can be resolved into nine distinct phylogroups (Ko1 to Ko9), which are believed to comprise six species (Merla et al., 2019). Phylogroups are based on specific beta-lactamase (*bla*_OXY__–(__1__–__9__)_) gene variants unique to this complex (Fevre et al., 2005; Merla et al., 2019) and each major phylogroup (except Ko5, Ko7 and Ko9) represents a unique species. Currently, *K. spallanzanii* (Ko3 or Ko9, *bla*_OXY–__3_ or *bla*_OXY–__9_) and *K. huaxiensis* (Ko8, *bla*_OXY–__8_) can be differentiated biochemically from other complex members (Merla et al., 2019). In contrast, a bacterial isolate identified as *K. oxytoca* by classical polyphasic typing strategies can actually be one of several species: *K. oxytoca* (Ko2, *bla*_OXY–__2_*), K. michiganensis* (Ko1 or Ko5, *bla*_OXY–__1_ or *bla*_OXY–__5_), *K. pasteurii* (Ko4, *bla*_OXY–__4_), or *K. grimontii* (Ko6, *bla*_OXY–__6_). Phylogroup Ko7 (*bla*_OXY–__7_) has been described based on a single strain and represents a sub-group of Ko6 (Izdebski et al., 2015).

Members of the KoSC are versatile pathogens and have been isolated from wound-, intra- abdominal-, urinary-, and lower respiratory tract infections (Herzog et al., 2014; Singh et al., 2016). Outbreaks have environmental sources (Hendrik et al., 2015) and subsequent dissemination of phylogroup members in healthcare settings is a major problem – especially in neonatal intensive care units (Rønning et al., 2019; Chapman et al., 2020; Chen et al., 2020). Outbreaks involve strains with extended-spectrum beta-lactamases (Lowe et al., 2012; Vasaikar et al., 2017), extended-spectrum activity of the chromosomal beta-lactamase (Fournier et al., 1995; Decré et al., 2004) and carbapenemases (Validi et al., 2016). Isolates can also carry resistance to fluoroquinolones and tetracyclines limiting therapeutic options (Brisse et al., 2000). KoSC members are also residents of the human gastrointestinal tract and under certain conditions act as pathobionts in adults and children. Intestinal enrichment with KoSC members is frequent in preterm neonates. *Klebsiella* overgrowth in infants due to antibiotic therapy is associated with late-onset sepsis, meningitis, and necrotizing enterocolitis, but the importance of cytotoxin production to disease is not yet clear (Carrie et al., 2019; Chen et al., 2020; Paveglio et al., 2020). Difficulties distinguishing these closely related complex members have not only led to inconsistent species classification in recent literature and databases but also make it impossible to draw conclusions regarding the clinical relevance of the individual species.

Here we report refined phylogenetic analyses of this closely related complex and delineate a uniform system of species classification that is equally valid for its monophyletic members.

## Methods

### Collection and Characterization of Environmental Strains

#### Sample Collection

Samples were taken from activated sludge (*n* = 29), surface water (*n* = 2), minced meat (*n* = 36), fresh retail chicken (*n* = 30), plant roots and soil (*n* = 31). The food was obtained from supermarkets and butcher shops in Austria. The activated sludge samples were collected from two different Austrian sewage treatment plants with a maximum load of <10,000 population equivalent (PE) and >100,000 PE, respectively. Plant roots and associated soil samples were taken from *Fabaceae*, *Pinaceae*, *Solanaceae*, *Rutaceae*, *Poaceae*, and *Vitaceae*.

#### Identification of *K. oxytoca*

Food samples were prepared according to the International Organization for Standardization, 2003 ISO 6887-2:2003. For enrichment 25 g of the meat samples were mixed with 225 ml peptone broth 1% (Oxoid Ltd., Basingsoke, England), homogenized, then cultured 16 to 24 h at 37°C under shaking. 1 g of plant roots and soil samples were mixed with 25 ml peptone broth 1% and enriched overnight. Activated sludge and surface water samples were screened without enrichment. Serial dilutions were cultured on chromID^TM^ CPS^®^ Agar (bioMérieux, Marcy-l’Etoile, France) for 24-48 h at 37°C. Colonies were scored based on the colour reaction of CPS media. Green to turquoise colonies were identified as *Klebsiella* spp., *Enterobacter* spp., *Serratia* spp*., Citrobacter* spp. and *Enterococcus* spp. pink to burgundy colonies as *E. coli* and light brown to brown colonies as *Proteus* spp. For pure cultures green to turquoise colonies were transferred to blood agar and Endo agar (24 h, 37°C). The isolate identification was carried out biochemically with VITEK^®^ 2 and/or with MALDI-TOF MS analysis, using VITEK^®^ MS (bioMérieux).

#### Genotypic and Phenotypic Toxin Assessment

All environmental isolates were screened for *npsB* presence and cytotoxicity. A 231 bp region of *npsB* was amplified by colony PCR using primer pair 1 + 2 (Table 1) in 30 cycles and primer annealing at 60°C. Cytotoxicity of supernatants of *K. oxytoca* spent medium was tested after 16 h culture. Viability of HeLa cells was used for the colorimetric 3-[4,5-dimethyl-2-thiazolyl] 2,5-diphenyl-2H-tetrazolium bromide (MTT) assay of mitochondrial reductase activity as previously described (Joainig et al., 2010).

**TABLE 1:** Oligonucleotides used in PCR-screening and sequencing.

#	Primer name	Sequence 5′-3′	Binding Site (nt position) in	Reference sequence	Fragment size	Reference
1	npsB_f	ctcgacgttttatctctgctg	20117..20137	AHC-6*	231 bp	This study
2	npsB_r	ttcctgaagtatctgccctgc	20327..20347			This study
3	OXY1-A	gtggcgtaaaaccgccctg	5001082..5001100	CAV1374	425 bp	Fevre et al., 2005
4	OXY1-B	gtccgccaaggtagctaatc	5001487..5001506			Fevre et al., 2005
5	OXY2-A	aaggctggagattaacgcag	4572112..4572131	NCTC13727	155 bp	Fevre et al., 2005
6	OXY2-B	gcccgccaaggtagccgatg	4572247..4572266			Fevre et al., 2005
7	OXY-E	ggttt**T**ggtaactgtgacggg	5304405..5304937	NCTC13727	1,098 bp	Fevre et al., 2005
8	OXY-G	cagagt**G**cagagtgttgcag	5304405..5304937			Fevre et al., 2005
9	core_f	gagatcccaagttctttagcaatgg	227821..227845	NCTC13727	175 bp	This study
10	core_r	agcacgttttccaggcgctgg	227976..227995			This study
11	leupA_f	atgaagatagcgattcacaac	315403..315425	CAV1374	1,235 bp	Li et al., 2020
12	leupB_r	gcgtggtcttttagctgttc	316620..316639			Li et al., 2020
13	orfA_f	gcagtgatttaaatactcttgcgg	53092..53115	JKo3	2,525 bp	This study
14	orfC_r	ccgatacctccagtaatgcgc	55617..55637			This study
15	B2_v2_1	cggcttacgcacaaagaagcc	74467..74487	ARO112, cont20	591 bp	This study
16	B2_v2_2	atgtttcttgaagaacgtagg	75037..75075			This study
17	gapA_fwd	gttttcccagtcacgacgttgtatgaagtatgactccactcacgg	4012172..4012193	CAV1374	680 bp	Herzog et al., 2014
18	gapA_rev	ttgtgagcggataacaatttcaacgcctttcattgcgccttcggaa	4012827..4012851			Herzog et al., 2014
19	infB_fwd	gttttcccagtcacgacgttgtactctctgctggactacattcg	1034476..1034496	CAV1374	463 bp	Herzog et al., 2014
20	infB_rev	ttgtgagcggataacaatttccgctttcagctccagaacttc	1024918..1034938			Herzog et al., 2014
21	mdh_fwd	gttttcccagtcacgacgttgtacccaactgccttcaggttcag	964459..964479	CAV1374	704 bp	Herzog et al., 2014
22	mdh_rev	ttgtgagcggataacaatttc**C**cttc**C**acgtaggcgcattcc	965141..965162			Herzog et al., 2014
23	pgi_fwd	gttttcccagtcacgacgttgtagagaaaaacctgccggtgctgctg	21866..21889	CAV1374	701 bp	Herzog et al., 2014
24	pgi_rev	ttgtgagcggataacaatttccggttaatcag**G**ccgttagtggagc	21189..21213			Herzog et al., 2014
25	phoE_fwd	gttttcccagtcacgacgttgtaacct**GG**cgcaa**C**accga**T**ttcttc	5304914..5304937	CAV1374	533 bp	Herzog et al., 2014
26	phoE_rev	ttgtgagcggataacaatttcttcagctggttgattttgtaatccac	5304405..5304430			Herzog et al., 2014
27	rpoB_fwd	gttttcccagtcacgacgttgtaggcgaaatggcggaaaacca	111644..111663	CAV1374	1,076 bp	Herzog et al., 2014
28	rpoB_rev	ttgtgagcggataacaatttcgagtcttcgaagttgtaacc	110588..110607			Herzog et al., 2014
29	tonB_fwd	gttttcccagtcacgacgttgtactctatacttcggtacatcaggtt	2890556..2890579	CAV1374	589 bp	Herzog et al., 2014
30	tonB_rev_2	ttgtgagcggataacaatttcgtttacccggttcatagcgcc	2891124..2891144			This study
31	tonB_rev	ttgtgagcggataacaatttccctgtttggcggccagcacctggt	2686207..2686230	NCTC13727**	740 bp	Herzog et al., 2014
32	MLST_seq_fwd	gttttcccagtcacgacgttgta	n/a	n/a	n/a	Herzog et al., 2014
33	MLST_seq_rev	ttgtgagcggataacaatttc	n/a	n/a	n/a	Herzog et al., 2014

*Tilimycin/tilivalline biosynthesis gene cluster (accession number: HG425356.1). **Only first 4 nucleotides on the 3′ end of the primer bind in CAV1374 in the first reading frame after tonB (in the fructosamine kinase family protein), therefore primer binding site is given for NCTC13727. underlined: binding site for MLST_seq primers; **bold:** primer binding site ambiguity in given reference seq.

#### Beta-Lactamase Gene Typing and Detection of Accessory Genes

Identification of beta-lactamase variants *bla_OXY–__1_* and *bla_OXY–__2_* utilized primer pairs 3 + 4 and 5 + 6, respectively, as previously described (Fevre et al., 2005) but with annealing at 55°C. The sequence of amplicons generated using oligonucleotides 7 and 8 determined all other *bla*_OXY_ variants. Amplification of *leupAB* utilized primers 11 + 12 (1,235 bp), of *orfABC* primers 13 + 14 (2,546 bp), and of *orfA*′ primers 15 + 16 (591 bp). Absence of an insertion was confirmed by colony PCR over the conserved insertion site with primer pair 9 + 10 (174 bp fragment). Reactions were performed in 35 cycles with 60°C annealing.

#### MLST and Phylogenetic Analysis

Isolates were typed as described (Herzog et al., 2014) using oligonucleotides listed in Table 1. Isolates that failed to produce a PCR product for *tonB* with primer pair 29 + 31 were reanalyzed using primer pair 29 + 30. Oligonucleotide 30 (tonB_rev_2) binds at the 3′ end of *tonB* in contrast to the originally published tonB_rev primer, which binds distally to the *tonB* reading frame. The PCR product thus obtained still allows analysis of the full allelic region used for typing. Oligonucleotides 32 and 33 were used for sequencing the resulting PCR products. New alleles, newly identified STs and all typed isolates were submitted to pubmlst.org/organisms/klebsiella-oxytoca. The concatenated sequence alignment of all MLST loci was used to infer the phylogenetic relationship among the strains analyzed in this study. The Neighbor-joining tree was built in MEGAX (Kumar et al., 2018) with Tamurai-Nei model and gamma distribution, and utilized *K. pneumoniae* for tree rooting (Herzog et al., 2014).

### Comparative Analysis of Whole Genome Sequences

#### Genome Extractions, Reference Set Compilation, and Validation

Workflow is illustrated in Supplementary Figure 1.

In June 2020, all available KoSC genomes independent of their assembly status were downloaded from NCBI GenBank and analyzed (*n* = 316). First, a subset of these genomes comprising clinical, animal, and environmental isolates were selected based on completeness and overall quality, with threshold set at 30 scaffolds maximum (Supplementary Table 1). Additional strains from recent publications were included independent of their assembly status (see section “Result” for further information) (Supplementary Table 1). The average nucleotide identities (ANI) for this subset (*n* = 62) were calculated using ANIm based on MUMmer (NUCmer) v.3.23 (Kurtz et al., 2004) and PYANI (Pritchard et al., 2016) was used for score visualization (Figure 3). Comparative phylogenetic analysis, beta-lactamase-, and accessory gene typing were then applied to these 62 strains to establish a robust reference data set (Figure 4). To validate our findings we assessed phylogeny, core gene and accessory gene distribution of all 316 available KoSC genomes independent of assembly status and together with 24 other *Klebsiella* genomes (Figure 5 and Supplementary Table 2). In a final step, we validated our findings by short read sequence typing of non-assembled KoSC genomes (*n* = 41) of clinical origin (NCBI BioProject PRJEB5065) (Moradigaravand et al., 2016, 2017).

**FIGURE 1:**
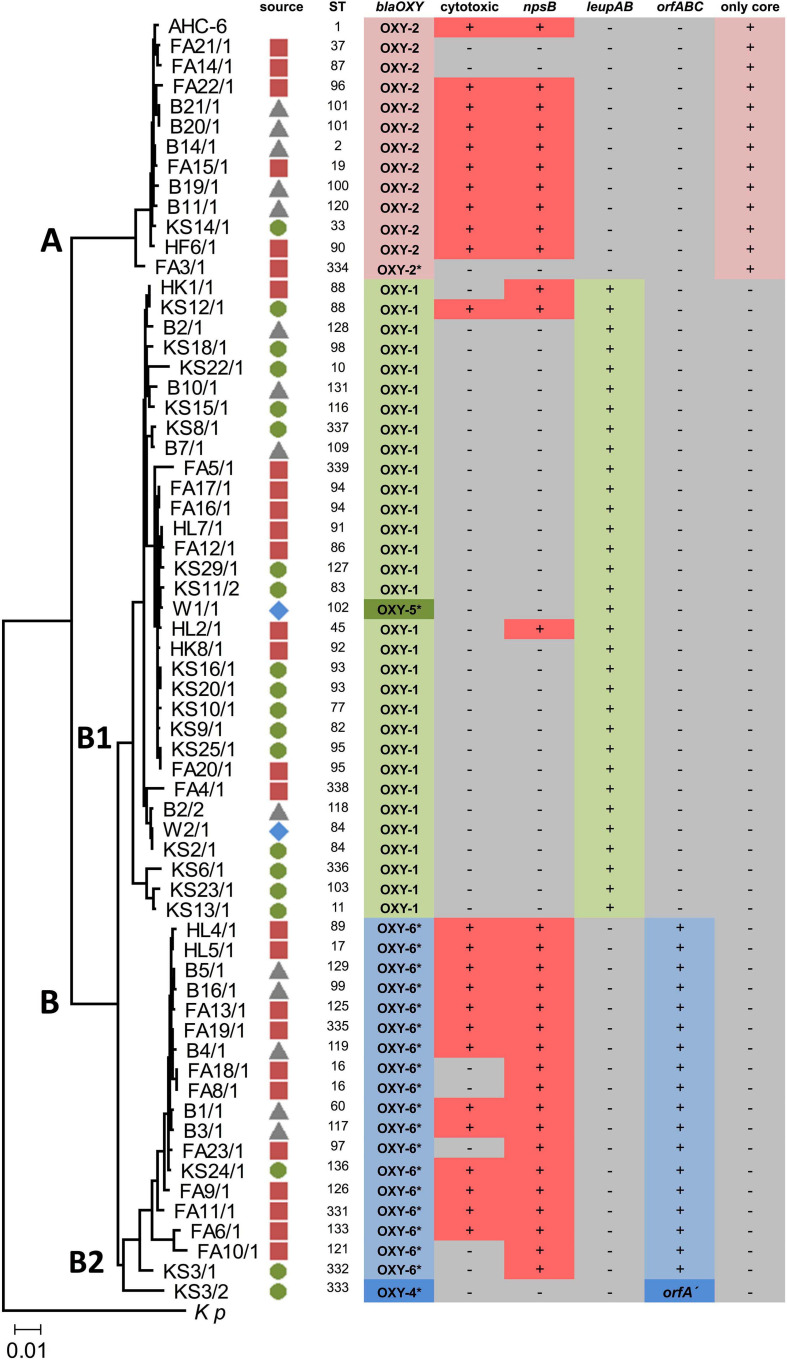
Neighbor-joining tree showing the genetic relatedness of 63 environmental isolates. Source symbols next to strain designation indicate isolation site (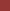 food; 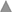 soil; 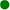 sewage sludge; 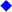 surface water). The tree is based on the concatenated sequences of seven housekeeping gene loci used in MLST. *K. pneumoniae* (*Kp*) was used for tree rooting. Clusters (A,B) and sub-clusters (B1,B2) are indicated. The scale bar represents 0.01 substitutions per site. MLST reference strain *K. oxytoca* AHC-6 (sequence type, ST1) is included (top). *bla*_OXY_ variants (synonymous to phylogroups) were determined using PCR and where noted (*) amplicon sequencing. Cytotoxicity was verified by MTT assays. Positive (+) and negative (-) PCR results for *npsB*, *leupAB*, *orfABC/orfA*′ or “only core” are indicated.

**FIGURE 2:**
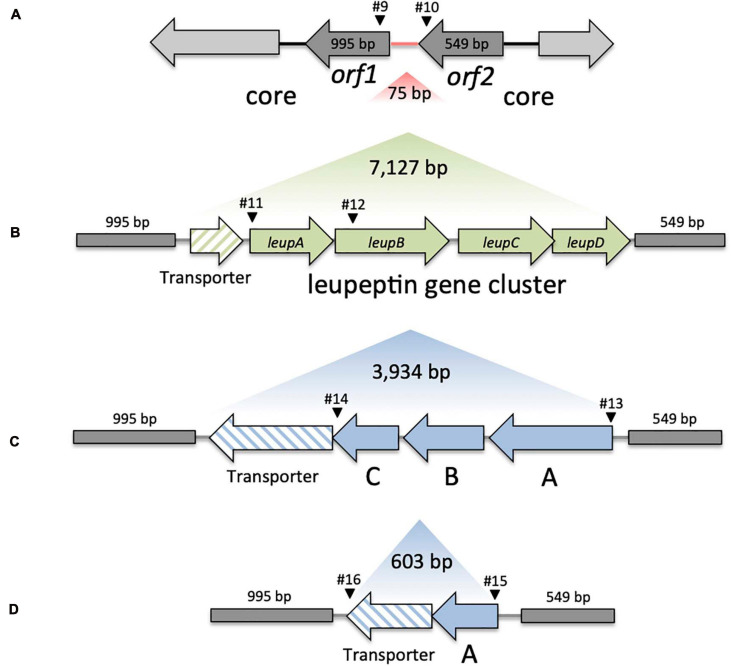
Genomic variation in KoSC strains. **(A)** Overview of the core region lacking insertion (Variant A). Region between *orf1* and *orf2* indicated in red represents the insertion site for panel **(B)** Variant B, the transporter and *leupABCD*, **(C)** Variant C, transporter and *orfABC* and **(D)** Variant D, the truncated transporter and truncated *orfA*′. Sizes of the region lacking insertion, core genes, and respective gene clusters are indicated in bp. Black arrowheads indicate approximate binding sites and primer numbers applied in PCR screens of environmental test set (Table 1).

**FIGURE 3:**
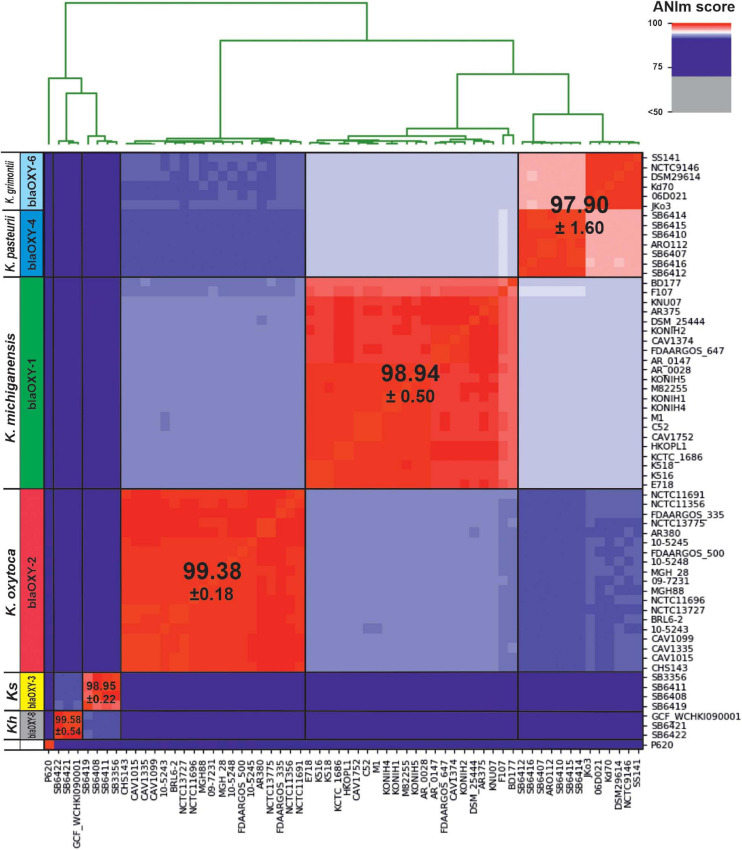
Average nucleotide identity (ANI) detects 5 distinct phylogenetic groups for the KoSC. Here we show genetic distance between 62 genome sequences affiliated with one of the complex species. Scores were calculated with ANIm based on MUMmer alignments and visualized using PYANI (Pritchard et al., 2016). Observed clustering clearly correlates with blaOXY-based phylogroups as indicated horizontally. Affiliated species designations are shown and the average ANI score for each group is indicated. *Ks, K. spallanzanii; Kh, K. huaxiensis*.

**FIGURE 4:**
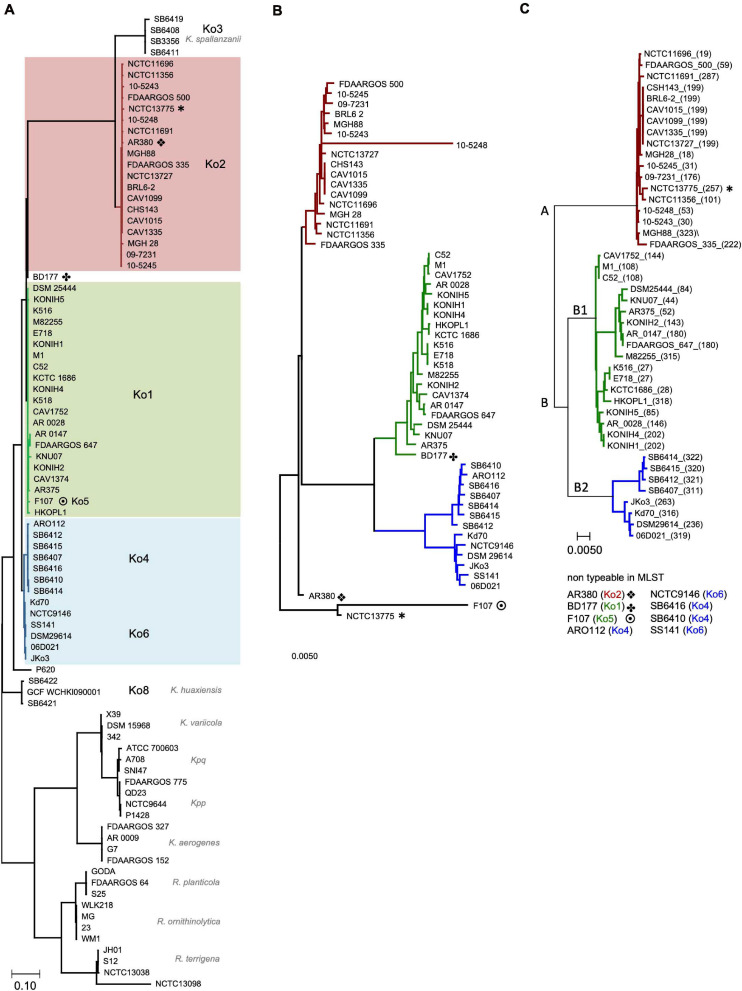
Phylogenetic analysis shows congruency of typing methods. **(A)** Phylogenetic tree inferred from concatenated, partitioned alignment of 136 marker proteins (Amphora2, supermatrix). Mayor tree clusters, which correspond to phylogroups (Ko), are highlighted in color (red, Ko2; green, Ko1; blue, Ko4 and Ko6). *K. spallanzanii* (Ko3), *K. huaxiensis* (Ko8) are indicated and species affiliation for each sub-branch of the out-group is shown; The out-group was used to root the tree. **(B)** Neighbor-joining tree based on the shared core genome of analyzed strains. Phylogenetic relationship was resolved based on 2,785 proteins. **(C)** Neighbor-joining tree based on 7 concatenated housekeeping gene loci used for MLST typing. Major MLST clusters (**A** and **B**) and sub-clusters B1 and B2 are indicated. Sequence types (STs) are given in parenthesis following the strain designations. Non-typeable strains are listed below with their respective phylogroup. Trees in panels **(B,C)** are mid-point rooted. Scale bars are indicated for each tree. Strains that do not cluster congruently between the trees are indicated by symbols. * is one of the symbols used to indicate incongruent clustering.

**FIGURE 5:**
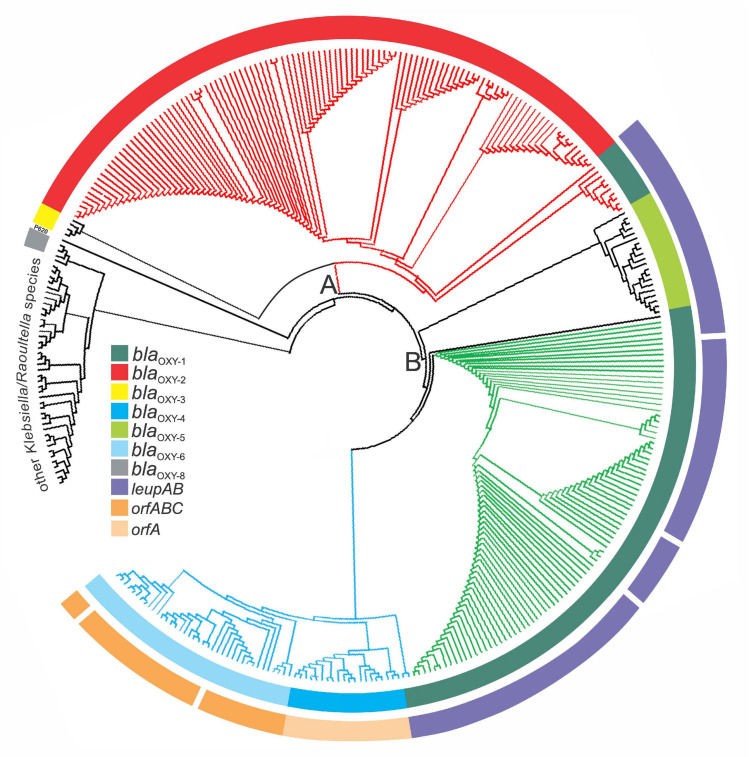
Phylogenetic representation of 341 *Klebsiella* genomes retrieved from GenBank. Phylogenetic analysis was done with PhyloPhlAn. The three main branches are colored according to MLST clusters and phylogroups as previously described: Cluster A, red; sub-cluster B1, green, sub-cluster B2, blue. The inner ring is colored based the respective *bla*OXY variant present (representing phylogroups Ko1-Ko8) and the outer ring indicates the presence or absence of species-specific marker genes for *K. michiganensis* (*leupAB*) and *K. grimontii* (*orfABC*) or *K. pasteurii* (*orfA*′). Other *Klebsiella*/*Raoultella* species: *K. huaxiensis* and 18 of 313 database entries of KoSC members clustered outside the major tree branches A and B with 24 reference strains (Supplementary Table 2).

#### Phylogenetic Analysis

Comparative phylogenetic analysis of whole genome sequences utilized three different approaches. (I) Phylogenetic analysis was done with PhyloPhlAn –version 3.0.51 (May 11, 2020) (Segata et al., 2013) using the amphora2 database (136 marker genes). The diversity was set to low and the default supermatrix configuration file was applied which utilizes five external tools: diamond, mafft, trimal, FastTreeMP, raxmlHPC.

(II) Core genome phylogeny of the biochemically identical KoSC members was created after extracting core genomes in the gbff format with the BPGA –version 1.3 (Chaudhari et al., 2016), with the sequence identity cut-off set at clustering 0.5 (50%). Core sequences were then aligned in MEGAX using MUSCLE with cluster method set as UPGMA. After initial alignment, the core tree was built in MEGAX. The construct was built using the Neighbor Joining method with Poisson model and gamma distribution set at one.

(III) Sequence types (STs) for GenBank entries were determined using pubmlst.org/organisms/klebsiella-oxytoca and a Neighbor-joining tree was built as described for the environmental strains except that the tree was midpoint rooted.

#### Blast Analyses and *in silico* PCR Analysis

*Klebsiella* genomes were analyzed with a standard online nucleotide Blast (query cover: 100%, *e*-value cut-off: 1e-30) using *leupABCD* as a query sequence. Screening of core genome insertion sites, genes and intergenic regions was done with blastn, where chromosomal and plasmid sequences for each strain were combined and built into a blast database. Assembly IDs of genome sequences used in blast analyses are listed in Supplementary Table 2.

Insertion site evaluation was performed with a blast query of 9,264 bp including *leupABCD*, the adjacent transporter and the two open reading frames (ORFs) flanking this region. The query sequence was retrieved from *K. oxytoca* CAV1374, nucleotide position 312527 to 321790. The five blastn outcomes obtained were defined as follows. Variant A: blast identities (>92.5%) for the ORFs flanking the leupeptin biosynthesis genes and no coding sequence in between; Variant B: blast identities (>94.64%) over the whole query sequence; Variant C: blast identity (>93.74%) for the flanking ORFs, separated approximately by 4,200 bp indicating an unknown insertion. Variant D: blast identity (>92.30%) for the flanking ORFs with an insertion of approximately 870 bp; Variant E: no significant identity to the query.

To compare PCR results from environmental samples with blast analysis of genomic data, we used the corresponding amplified gene fragment as the query sequence in blastn analysis, except for a full-length *npsB* query. Only results with homologies of >98.5% (*leupAB*, *orfABC*, *orfA’*) or >93,5% (*npsB*) over the full query sequence were scored as positive. Strains were also analyzed for their *bla*_OXY_ variants using each full-length gene sequence as a query. We performed *in silico* PCR analysis for *leupAB*, *orfABC*, and *orfA’* on whole genome sequences using a perl script and 100% primer binding stringency or allowed one base mismatch per primer sequence (Supplementary Table 2).

#### Short Read Sequence Mapping

Raw sequencing reads of 41 KoSC strains and 30 non-KoSC strains (2 *Acinetobacter baumannii*, 2 *Escherichia coli*, 3 *K. pneumoniae*, 7 *K. quasipneumoniae*, 2 *Klebsiella spp*. and 14 *K. variicola*) were downloaded from NCBI BioProject PRJEB5065 (Moradigaravand et al., 2016, 2017). Short Read Sequence Typing for Bacterial Pathogens (SRST2) (Inouye et al., 2014) was utilized for read-mapping (fastq format) and allele calling. As query sequences we used the same gene fragments (*leupAB*, *orfABC*, and *orfA’*) utilized for blast-based gene typing. To identify the conserved core genome site without variant insertion, we utilized the 175 bp fragment generated with primers 9 + 10. Full-length genes were used for *npsB* and variant *bla*_OXY__1–9_ calling. Default parameters were used with coverage threshold set at 90. Results were manually evaluated and hits with >99% coverage and >0.90 identity score were determined positive and included in Supplementary Table 3. For alleles with multiple hits (i.e., blaOXY) the highest homology variant is shown.

#### Relevant Assembly IDs and Accession Numbers

Assembly IDs of genomes used in this study can be found in Supplementary Table 2. Accession numbers of *bla*_OXY_ variants: AJ871864 (*bla*_OXY–__1_), AJ871866 (*bla*_OXY–__2_), AF491278 (*bla*_OXY–__3_), AY077481 (*bla*_OXY–__4_), AJ871871 (*bla*_OXY–__5_), AJ871875 (*bla*_OXY–__6_); KT001254.1 (*bla*_OXY–__7_);  (*bla*_OXY–__8_); and MN030564.1 (*bla*_OXY–__9_); Non-ribosomal peptide synthetase gene (*npsB*) of the *K. oxytoca* AHC-6 tilimycin/tilivalline biosynthesis gene cluster (HG425356.1) nucleotide position 17,602 to 21,972. Leupeptin biosynthesis gene cluster (nucleotide position 315,353 to 321,183) of strain CAV1374 (CP011636.1); locus tags for *leupA* (AB185_06775), *leupB* (AB185_06780), *leupC* (AB185_06785), and *leupD* (AB185_06790). *OrfABC* (nucleotide position 52,982 to 56,916) of strain JKO3 (AP014951.1); locus tags for *orfA* (KOJKO3_c0051), *orfB* (KOJKO3_c0052), *orfC* (KOJKO3_c0053), and the transporter (KOJKO3_c0054). *OrfA*′ and transporter (nucleotide position 74,455 to 75,057) of strain ARO112 (GCA_009757395.1, contig20), locus tags for *orfA*′ (GQ640_RS25055) and the transporter (GQ640_RS24900).

## Results

### Core Phylogeny and Accessory Gene Content Are Highly Congruent for a Population of Environmental KoSC Isolates

To explore the presence of phylogroup-specific genes in a diverse population we first characterized a collection of 63 KoSC strains isolated from various environmental sources (Figure 1). Identification was performed by routine biochemical analyses and MALDI-TOF mass spectrometry. We then genotyped the strains’ chromosomally encoded *bla*_OXY_ variants (Figure 1). PCR typing for *bla*_OXY–__1_ and *bla*_OXY–__2_ was sufficient, but reliable assessment of other *bla*_OXY_ variants required sequencing. Most strains were assigned to phylogroups Ko1 (*bla*_OXY–__1_), Ko2 (*bla*_OXY–__2_), and Ko6 (*bla*_OXY–__6_). A single Ko5 and one Ko4 (*bla*_OXY–__4_) isolate were also identified. Thus, we conclude that our collection comprised *K. oxytoca* (*bla*_OXY–__2_), *K. michiganensis* (*bla*_OXY–__2_ & *bla*_OXY–__5_), *K. pasteurii* (*bla*_OXY–__4_), and *K. grimontii* (*bla*_OXY–__6_) strains.

A multi-locus sequence typing (MLST) scheme has been developed for *K. oxytoca* (Herzog et al., 2014) that also resolves phylogeny of *K. michiganensis* (*bla*_OXY–__1_), *K. pasteurii* (*bla*_OXY–__4_) and *K. grimontii* (*bla*_OXY–__6_) (Fevre et al., 2005; Izdebski et al., 2015; Moradigaravand et al., 2017). However, correspondence of blaOXY grouping and MLST typing has not been explored extensively. We applied MLST to all isolates to compare with blaOXY-based phylogrouping and to investigate the diversity of our collection. Allelic variation at seven housekeeping gene loci identified 57 distinct sequence types (ST). Several of the STs match STs previously identified for clinical isolates. We then inferred a phylogenetic tree for this population and in good agreement with the population structure of clinical isolates (Herzog et al., 2014; Moradigaravand et al., 2017), relationships of the environmental strains cluster into two major clades (MLST-cluster A and B). Cluster B further splits into the sub-groups B1 and B2 (Figure 1). In summary, we obtained correspondence of MLST based phylogeny and blaOXY typing in this diverse population.

We next surveyed the population for accessory gene content. Knowledge of whether human KoSC isolates carry genes for production of the enterotoxins tilimycin and tilivalline (Dornisch et al., 2017; Unterhauser et al., 2019) is clinically relevant. Application of a PCR-based screen for the presence of a non-ribosomal peptide synthetase gene (*npsB*) to this collection showed that *npsB* affiliated predominately with cluster A and sub-group B2 of this population but was also present in B1 (Figure 1). All isolates negative for PCR product were also negative phenotypically for toxin production, but from 30 *npsB* + isolates, 7 were not cytotoxic (Figure 1). The observed general distribution of *npsB* effectively disqualified the locus as a phylogroup-specific marker.

Previously, we observed that the biosynthesis genes (*leupAB*) of protease inhibitor leupeptin (Li et al., 2020) have a characteristic distribution along MLST-based KoSC phylogeny. We used PCR to screen the collection for *leupAB* and consistent with our previous findings (Li et al., 2020), leupeptin biosynthesis genes were detected solely in strains of sub-cluster B1 and thus only in *K. michiganensis* (*bla*_OXY–__1_). To interrogate the correlation between leupeptin biosynthesis genes and *K. michiganensis* in more detail, we performed an online blastn search using *leupABCD* as a query sequence and “*Klebsiella”* as search set. The locus was detected in whole genome sequences classified as *K. oxytoca* and *K. michiganensis* but, concordant with our findings for the environmental collection, these genomes all carry *bla*_OXY__–__1_ suggesting a misclassification of database entries. To obtain better discrimination we surveyed the insertion site of *leupABCD.* We noted that the position is conserved in every *leupABCD* positive genome; therefore we next asked whether *leupABCD* negative strains carry distinct gene loci at this site. We utilized a >9kb query sequence comprising the conserved flanking open reading frames (*orf1* and *orf2*; Figure 2A) and spanning the leupeptin biosynthesis genes (Figure 2B) for blast analysis. We sought to focus specifically on *K. oxytoca* and performed blast analysis and blaOXY typing only on database entries with the identifier “*K. oxytoca*” available in NCBI (*n* = 177, June 2020). Five different outcomes were obtained for the genome region of interest (Figure 2). Strains either carry (I) the conserved core genes without insertion (variant A), (II) the conserved core genes with the leupeptin biosynthesis genes inserted (variant B), (III) the conserved core genes and a gene cluster we designated here as *orfABC* (variant C), (IV) core genes and a truncated version of the *orfABC* locus, *orfA*′ (variant D), or (V) no homology to the query. Importantly, despite our aim to analyze exclusively genomes belonging to the species *K. oxytoca*, we identified strains of all phylogroups. We next asked whether each insertion variant correlated with a specific blaOXY phylogroup. In 176 of the 177 genomes we observed perfect correlation between the two loci (Supplementary Table 2). Variant A strains (no insertion) carry *bla*_OXY__–__2_, variant B strains (leupeptin biosynthesis genes) carry *bla*_OXY__–__1_ or *bla*_OXY__–__5_, variant C strains (*orfABC*) carry *bla*_OXY__–__6_, and the single observed variant D strain (*orfA*′) was positive for *bla*_OXY__–__4_. Only *K. oxytoca* strain K3678 represents an exception (*bla*_OXY__–__1_ but no leupeptin genes).

Based on these findings we applied a discriminatory PCR screen using primer-binding sites shown in Figure 2 to survey the alternate accessory gene sets carried by the environmental isolates (Figure 1). All B1 strains (including the *bla*_OXY–__5_ isolate) contain *leupAB* (variant B) and all but one B2 isolate carry *orfABC* (variant C). This *bla*_OXY–__4_ isolate (KS3/2) harbored the truncated *orfA*′ (variant D). All cluster A strains were negative for *leupAB*, *orfABC*, and *orfA*′ and amplicons from primers binding in the core genes confirmed that the region lacked insertion (variant A). Sequence similarity search with the newly identified *orf*A, *orfB*, and *orfC* and subsequent analyses of the conserved domains they contain imply a role in carbohydrate metabolism.

Results summarized in Figure 1 show that core genome phylogeny, *bla*_OXY_ variations, and accessory gene distribution correspond in this population. Thus, we conclude that not only variations in the core genome differentiate the species of the KoSC but also species-specific gene loci. We hypothesize that the discrepancies observed in correlating *bla*_OXY_ and accessory gene markers with species classification in database entries may result from misclassification. We further propose that the robustness of these correlations should facilitate typing and re-typing of isolates and will also consolidate species boundaries within the complex.

### Average Nucleotide Identity Reveals Discrepancies in Standing *K. oxytoca*, *K. michiganensis*, and *K. grimontii* Classification

Inconsistencies in taxonomic assignment of deposited sequences of *K. oxytoca* has been noted by others previously (Chen et al., 2020) and the observed incongruence in classification is not unexpected as some strains were classified before the phylogroups were separated into species. However, also recent database submissions are misidentified. The problem is perpetuated by incorrectly classified references strains whose use confounds consistent taxonomy of KoSC members.

To move forward, we thought to provide a verified reference set of consistently classified KoSC members. We performed an in-depth analysis of all KoSC genomes retrieved from databases and applied a comparative approach to develop the discriminatory power of typing methods. A survey of database entries deposited for KoSC members retrieved none with the identifier “*K. spallanzanii*” or “*K. pasteurii*” and only 4 sequences of *K. huaxiensis*. We then downloaded all publicly available genome sequences for *K. oxytoca*, *K. michiganensis*, *K. grimontii* and *K. huaxiensis* and identified four *K. spallanzanii* genomes (*bla*_OXY__–__3_) and 20 *K. pasteurii* (*bla*_OXY__–__4_) among them. To generate a robust test set (Supplementary Table 1), we selected all genomes with complete status. To cover phylogroups more evenly we also enrolled genomes with more than 30 contigs where necessary. We further added the following strains from recent publications regardless of their assembly status: *K. oxytoca* strain DSM29614 (Gallo et al., 2018), *K. grimontii* reference strain 06D021 (Passet and Brisse, 2018), the identified *K. spallanzanii* strains (Merla et al., 2019), *K. michiganensis* strain Kd70 (Dantur et al., 2018), *K. michiganensis* ARO112 (Oliveira et al., 2020), and reference strain DSM25444, which was the first *K. michiganensis* strain described (Saha et al., 2013).

Average nucleotide identities (ANI) of a large set of KoSC members has not been reported, thus we chose to first evaluate species boundaries in the KoSC test set assembled above using this unbiased whole-genome similarity metric regardless of the existing taxonomy. ANI resolved clusters in 5 major groups (Figure 3). blaOXY group and accessory gene typing of each test strain strongly correlated with the observed ANI affiliation (Figure 3 and Supplementary Table 1). *K. pasteurii* (*bla_OXY–__4_*) and *K. grimontii* (*bla_OXY–__6_*) are highly related. These are not differentiated when the ANI-based > 5% sequence discontinuity threshold for species separation is applied. Instead, a sub-clustering correlating with the respective blaOXY group is apparent. In summary, the average identity for *K. oxytoca* (*bla*_OXY–__2_) was 99.38 (SD ± 0.21), for *K. michiganensis*, (*bla*_OXY–__1_) 98.94 (SD ± 0.56), and for *K. grimontii* (*bla*_OXY–__6_) and *K. pasteurii* (*bla*_OXY–__4_) combined 97.90 (SD ± 0.94). Scores for the limited number of analyzed *K. spallanzanii* (*bla*_OXY–__3_) were 98.95 (SD ± 0.56), and for *K. huaxiensis* (*bla*_OXY–__8_) 99.58 (SD ± 0.56). Inter-group comparisons in this analysis support monophyly of the four biochemically identical species as they are more closely related to each other than to *K. spallanzanii* and *K. huaxiensis* (Figure 3). The putative *K. oxytoca* strain P620 was not typeable for blaOXY and is phylogenetically distant to all investigated phylogroups.

Next, we asked whether species separation can also be achieved using discrete sets of core genes. Robust phylogenetic trees were generated for the same test set of KoSC genomes using a variety of comparisons. The first tree (Figure 4A) is based on coding information for 136 reference proteins (Amphora2, supermatrix) and included as an outer-group other closely related *Klebsiella*/*Raoultella* species. The resulting tree clusters showed excellent agreement with ANI results. However, in contrast to ANI, this analysis based on selected reference proteins suggests a close relationship of Ko2 (*K. oxytoca*) and Ko3 (*K. spallanzanii*) strains. Notably, the assessment also shows that strains *R. ornithinolytica* S12 and *K. aerogenes* NCTC6944 should be reassigned to *R. terrigena* and *K. pneumoniae*, respectively. This classification was validated by ANI.

Finally, we generated phylogenetic trees for the biochemically identical species based on a large portion of the core genome (2,785 proteins) *versus* just seven conserved MLST core genes (Figures 4B,C). The comparison resulted in identical clustering except for strains AR380, BD177, F107, and NCTC13775. Notably, not all strains can be typed using MLST particularly *bla*_OXY–__4_ and *bla*_OXY–__6_ strains.

The comparison confirmed converging outcomes using multiple typing methods based on a wide spectrum of markers. The relevant conclusion is that MLST cluster A represents *K. oxytoca*, sub-group B1, *K. michiganensis*, and sub-group B2 comprises *K. pasteurii*, and *K. grimontii*, which, nonetheless could be clearly separated into distinct groups in every analysis performed. Thus, the phylogenies determined from STs of this reference set of genomes (Figure 4C and Supplementary Table 1) as well as the STs of the environmental test set (Figure 1) can provide a standard for species identification of KoSC isolates where MLST results are available.

### Unifying Classification Scheme for Distinction of the Biochemically Identical KoSC Members

We next performed a validation test by analyzing and visualizing the relationship of all 316 KoSC genomes including *K. spallanzanii* and *K. huaxiensis* and closely related *Klebsiella* and *Raoultella* species available in June 2020 (Supplementary Table 2). The inferred phylogenetic tree (Amphora2, supermatrix) showed a clustering in three major branches (Figure 5). Next, we interrogated the correlation of branching with presence of blaOXY variants and accessory gene markers. Blast analysis using *bla*_OXY_ variants, *leupAB*, *orfABC*, and *orfA*′ as query sequences established correlation of main clusters and phylogroups as well as with the MLST-tree branches A, B1, and B2 (Figure 5 and Supplementary Table 2). In good agreement with previous findings (Li et al., 2020), Ko5 (*bla*_OXY–__5_) strains cluster in branch B as a distinct group (intermediate cluster) but isolates share the *leupAB* locus present in sub-group B1 strains. Interestingly, we identified a selection of Ko1 (*bla*_OXY__–__1_) strains within cluster A. These isolates were positive for *leupAB* and therefore share features with sub-group B1 strains. According to ANI, these intermediate isolates are *K. michiganensis* strains and not *K. oxytoca* strains. Similarly, some *K. michiganensis* strains were affiliated with sub-group B2, yet they still carry *leupAB* and not *orfABC* or *orfA*′. This finding highlights the monophyletic relationship of these complex members as phylogenetic trees inferred from concatenated core genes cannot always fully resolve phylogroup boundaries. However, species separation is achieved based on a combination of blaOXY and accessory gene typing.

In summary, we argue that the results of the numerous approaches taken here provide sufficient evidence to support reclassification of 94 of 316 KoSC strains in the databases (Supplementary Table 2). Notably, 18 isolates did not cluster in any of the major branches and represent other closely related *Klebsiella*/*Raoultella* species (Supplementary Table 2).

### Validation of Rapid, Reliable Species Identification

To test the capacity for rapid reliable typing using the accessory gene content described above, we performed *in silico* PCRs on this validation set of all 316 KoSC genomes and the 24 other *Klebsiella* genomes (Supplementary Table 2) with the primer pairs used to detect *leupAB*, *orfABC*, and *orfA*′ in the environmental test set. Results were in good agreement with the outcome of blast analyses for the respective gene loci (Supplementary Table 2). Application of *in silico* PCR without allowing any mismatch detected *leupAB* in 89% (103/116) and, when only one mismatch per primer was allowed, in 95% (109/116) of the *K. michiganensis* genomes previously positive in *leupAB* blast analysis. With both primer stringencies *orfA’* was detected in 75% (15/20) of the *K. pasteurii* genomes previously positive in *orfA’* blast analysis and in 100% (38/38) of *K. grimontii* genomes previously positive in *orfABC* blast analysis. These *in silico* results confirm that primer-binding sites are highly conserved. Since we were able to type all environmental isolates experimentally with this PCR, we predict that the actual detection rate in practice will be higher than observed in the very stringent *in silico* PCR. Moreover, specificity appears to be given as *in silico* PCRs and blast analyses for the gene loci were negative for *K. spallanzanii*, *K. huaxiensis*, and other members of the *Klebsiella* group tested in this analysis (Supplementary Table 2).

In a final step, we verified the validity of our approach using non-assembled genome sequences. Fastq files of phylogenetically characterized KoSC strains of clinical origin (Moradigaravand et al., 2017) were extracted from BioProject PRJEB5065 (Moradigaravand et al., 2016) and subjected to short read sequence typing. Analysis of this population (Validation set II: clinical) revealed that the characteristic distribution of accessory genes along the KoSC phylogeny is also conserved in strains of clinical origin. In agreement with previously published data (Moradigaravand et al., 2017) we identified the respective blaOXY alleles for each strain as well as the phylogroup-matching accessory genes (Supplementary Table 3) *leupAB*, *orfABC*, or the genomic core region lacking any insertion. No *K. pasteurii* (*orfA’*) was identified in this strain set. All of the tested non-KoSC genomes lack *leupAB*, *orfABC*, *orfA’*, and *bla*_OXY_ variants. One exceptional *K. quasipneumoniae* strain gave a positive result for the conserved “empty” core region but the isolate was not positive for any of the *bla*_OXY_ variants.

Based on these analyses, we conclude that simple assessment of four gene loci can be applied for reliable species affiliation of monophyletic members of the KoSC. In this classification scheme defining criteria for differentiation of the biochemically identical species are: *K. oxytoca* (*bla*_OXY–__2_, *leupAB*-, *orfABC*-, *orfA*′-), *K. michiganensis* (*bla*_OXY–__1__&__5_, *leupAB* +, *orfABC*-, *orfA*′-), *K. pasteurii (bla*_OXY–__4_, *leupAB*-, *orfABC*-, *orfA*′+) and *K. grimontii* (*bla*_OXY–__6_, *leupAB*-, *orfABC*+, *orfA*′-).

## Discussion

Consistent, systematic classification of bacteria is challenging but highly important since nomenclature influences the way data gathered on organisms is interpreted. The KoSC comprises several closely related species of clinical and economic significance. To date *K. oxytoca, K. michiganensis, K. pasteurii* and *K. grimontii* cannot be distinguished morphologically, biochemically, or based on their 16s rRNA gene sequence. Moreover, they seem to inhabit the same ecological niches and share important virulence properties like the tilimycin/tilivalline biosynthetic gene cluster.

In this study we aimed to develop a uniform classification scheme with the capacity to define species of the KoSC with high resolution. We took a multi-pronged approach to determine the population structure of an uncharacterized collection of KoSC members of environmental origin. We applied genome-based species definition to the large volume of publicly available genomes. Congruency of phylogenies derived when indexing core and accessory genes helped validate genotypic relationships within the complex and also define clear genetic discontinuities. The data reveal a simple routinely applicable classification scheme with sufficient discriminatory power to resolve the biochemically identical *K. oxytoca*, *K. michiganensis*, *K. pasteurii* and *K. grimontii*. The data also expose discrepancies between the results of genome-based species definitions and standing nomenclature of the NCBI taxonomy database (Supplementary Table 2). Although stability in bacterial nomenclature is certainly desirable, reclassification of strains in this complex appears necessary. The community may choose to reclassify prior entries and possibly reinterpret clinical data gathered on organisms in the past. Such steps would help prevent continued confusion about organisms of the KoSC and clarify the clinical relevance of each species.

Further information gained from this study included insights into the distribution of KoSC members in the environment. We sought to recover strains from sewage sludge, soil, meat and surface water and ultimately cultured and isolated members of the complex from 68 of 128 environmental samples. Although the population size is too small to draw broad conclusions regarding environmental distribution of *K. oxytoca*, *K. michiganensis, K. pasteurii* and *K. grimontii* it is interesting to note that every second meat product analyzed was contaminated with at least one KoSC strain. Of these 32 isolates 19 were *npsB*+, implicating retail meat as a likely source of transmission of cytotoxin-producing *Klebsiella spp*. (Figure 1). Antibiotic-associated hemorrhagic colitis is caused by the overgrowth of toxigenic strains following use of antibiotics such as penicillin or amoxicillin (Högenauer et al., 2006). Transmission of toxin positive strains from food during antibiotic therapies might therefore increase patient risk for severe infections.

*Klebsiella michiganensis* strains can be isolated from the gastro-intestinal tract (Chen et al., 2020), blood and cerebrospinal fluid (Carrie et al., 2019) of infants, and they are affiliated with upper-respiratory diseases in adults (Herzog et al., 2014). In this study, we isolated *K. michiganensis* strains predominantly from sewage sludge. Previously, it was shown that sub-group B1 strains have the potential to synthesize protease inhibitor leupeptin, which might provide a colonization advantage in the lung (Li et al., 2020).

*Klebsiella grimontii* strains have been isolated from the gastro-intestinal tract of infants, adults, and animals as well as from diverse environmental sites (Passet and Brisse, 2018; Chen et al., 2020; Oliveira et al., 2020). Our analyses show that these *K. grimontii* strains include *K. pasteurii* (*bla*_OXY–__4_) isolates. Based on the ANI threshold 95, our analysis does not support a separation of phylogroups Ko4 and Ko6 strains into distinct species but strains of the two groups can clearly be differentiated based on core and accessory genes investigated in this study.

In summary, we show convergence of ANI grouping, blaOXY typing, and MLST phylogeny, thus previously obtained MLST data can be reinterpreted for species delineation. Routine laboratories will continue to identify strains following a conventional polyphasic strategy but resolution of that approach can now be improved with the rapid and simple PCR-typing strategy described here. The method allows robust species delineation without requiring whole genome sequencing or even *bla*_OXY_ sequencing. The available data allow us to propose that once an isolate is biochemically identified as *K. oxytoca*, PCR for *bla*_OXY–__1_ *and bla*_OXY–__2_ can reliably differentiate *K. michiganensis* (except *bla*_OXY–__5_) and *K. oxytoca* strains. Differentiation of *K. grimontii* (*bla*_OXY__–__6_) and *K. pasteurii* (*bla*_OXY–__4_) or identification of *bla*_OXY–__5_ strains requires *bla*_OXY_ gene sequencing. Yet, as an alternative in those cases, a simple PCR screen for *leupAB*, *orfABC*, and *orfA*′ is sufficient to differentiate the species.

## Data Availability Statement

The original contributions presented in the study are included in the article/Supplementary Material, further inquiries can be directed to the corresponding author/s.

## Author Contributions

EL, EZ, and SK conceived and designed the study. CP, HG, FR, GF, and CH conceived the environmental sampling. EL, CP, HG, KH-O, ET, SR, and SK isolated and characterized the environmental isolates. AC and SK collected and analyzed the data and performed the computational analysis. AC, EL, EZ, and SK wrote the manuscript. All authors contributed to the article and approved the submitted version.

## Conflict of Interest

The authors declare that the research was conducted in the absence of any commercial or financial relationships that could be construed as a potential conflict of interest.

## Supplementary Material

Supplementary Figure 1 Illustration of workflow. Five specific sets of samples/sequence data were sequentially analyzed **(A–E)**. Their respective isolation and/or download source is indicated above. The main selection criteria for compiling the respective data sets can be found together with the number of strains/genomes analyzed in the blue boxes. Orange boxes identify which analysis/tool was performed/applied on each data set and lead over to the green boxes that indicate which figure/table presents the respective results. A more detailed description of data sets and methodology is provided in the “Methods” section.

Supplementary Table 1 Summary of MLST and Blast results for the reference set of *Klebsiella oxytoca* species complex.

Supplementary Table 2 Summary of Blast and in silico PCR results for 316 *K. oxytoca* species complex members and 24 other *Klebsiella/Raoultella* species.

Supplementary Table 3 Summary of short read sequence typing of KoSC isolates.

## References

[B1] Brisse S. Milatovic D. Fluit A. C. Verhoef J. Schmitz F.-J. (2000). Epidemiology of Quinolone Resistance of *Klebsiella pneumoniae* and *Klebsiella oxytoca* in Europe. *Eur. J. Clin. Microbiol. Infect. Dis.* 19 64–68. 10706185 10.1007/s100960050014

[B2] Carrie C. Walewski V. Levy C. Alexandre C. Baleine J. Charreton C. (2019). *Klebsiella pneumoniae* and *Klebsiella oxytoca* meningitis in infants. *Epidemiol. Clin. Features. Archiv. Pédiatrie* 26 12–15. 10.1016/j.arcped.2018.09.013 30558858

[B3] Chapman P. Forde B. M. Roberts L. W. Bergh H. Vesey D. Jennison A. V. (2020). Genomic Investigation Reveals Contaminated Detergent as the Source of an Extended-Spectrum-β-Lactamase-Producing *Klebsiella michiganensis* Outbreak in a Neonatal Unit. *J. Clin. Microbiol.* 58 e1980–e1919. 10.1128/JCM.01980-19 PMC7180233 32102855

[B4] Chaudhari N. M. Gupta V. K. Dutta C. (2016). BPGA- an ultra-fast pan-genome analysis pipeline. *Sci. Rep.* 6:24373. 27071527 10.1038/srep24373 PMC4829868

[B5] Chen Y. Brook T. C. Soe C. Z. O’Neill I. Alcon-Giner C. Leelastwattanagul O. (2020). Preterm infants harbour diverse *Klebsiella populations*, including atypical species that encode and produce an array of antimicrobial resistance- and virulence-associated factors. *Microbial. Genomics* 6:377. 10.1099/mgen.0.000377 PMC7371107 32436839

[B6] Dantur K. I. Chalfoun N. R. Claps M. P. Tórtora M. L. Silva C. Jure Á, et al. (2018). The Endophytic Strain *Klebsiella michiganensis* Kd70 Lacks Pathogenic Island-Like Regions in Its Genome and Is Incapable of Infecting the Urinary Tract in Mice. *Front. Microbiol.* 9:1548. 10.3389/fmicb.2018.01548 30061870 PMC6054940

[B7] Decré D. Burghoffer B. Gautier V. Petit J.-C. Arlet G. (2004). Outbreak of multi-resistant *Klebsiella oxytoca* involving strains with extended-spectrum β-lactamases and strains with extended-spectrum activity of the chromosomal β-lactamase. *J. Antimicrob. Chemother.* 54 881–888. 10.1093/jac/dkh440 15472005

[B8] Dornisch E. Pletz J. Glabonjat R. A. Martin F. Lembacher-Fadum C. Neger M. (2017). Biosynthesis of the Enterotoxic Pyrrolobenzodiazepine Natural Product Tilivalline. *Angew Chem. Int. Ed.* 56 14753–14757. 10.1002/anie.201707737 28977734 PMC5698749

[B9] Fevre C. Jbel M. Passet V. Weill F.-X. Grimont P. A. D. Brisse S. (2005). Six Groups of the OXY β-Lactamase Evolved over Millions of Years in *Klebsiella oxytoca*. *AAC* 49 3453–3462. 10.1128/aac.49.8.3453-3462.2005 16048960 PMC1196214

[B10] Fournier B. Lu C. Y. Lagrange P. H. Krishnamoorthy R. Philippon A. (1995). Point mutation in the pribnow box, the molecular basis of beta- lactamase overproduction in *Klebsiella oxytoca*. *Antimicrob. Agents Chemother.* 39 1365–1368. 10.1128/aac.39.6.1365 7574532 PMC162743

[B11] Gallo G. Presta L. Perrin E. Gallo M. Marchetto D. Puglia A. M. (2018). Genomic traits of *Klebsiella oxytoca* DSM 29614, an uncommon metal-nanoparticle producer strain isolated from acid mine drainages. *BMC Microbiol.* 18:198. 10.1186/s12866-018-1330-5 30482178 PMC6258164

[B12] Hendrik T. C. Voor in ‘t holt A. F. Vos M. C. (2015). Clinical and Molecular Epidemiology of Extended-Spectrum Beta-Lactamase-Producing *Klebsiella spp*.: A Systematic Review and Meta-Analyses. *PLoS One* 10:e0140754. 10.1371/journal.pone.0140754 26485570 PMC4617432

[B13] Herzog K. A. T. Schneditz G. Leitner E. Feierl G. Hoffmann K. M. Zollner-Schwetz I. (2014). Genotypes of *Klebsiella oxytoca* Isolates from Patients with Nosocomial Pneumonia Are Distinct from Those of Isolates from Patients with Antibiotic-Associated Hemorrhagic Colitis. *J. Clin. Microbiol.* 52 1607–1616. 10.1128/jcm.03373-13 24599976 PMC3993621

[B14] Högenauer C. Langner C. Beubler E. Lippe I. T. Schicho R. Gorkiewicz G. (2006). *Klebsiella oxytoca* as a Causative Organism of Antibiotic-Associated Hemorrhagic Colitis. *N. Engl. J. Med.* 355 2418–2426. 17151365 10.1056/NEJMoa054765

[B15] Inouye M. Dashnow H. Raven L.-A. Schultz M. B. Pope B. J. Tomita T. (2014). SRST2: Rapid genomic surveillance for public health and hospital microbiology labs. *Genome Med.* 6:90. 25422674 10.1186/s13073-014-0090-6 PMC4237778

[B16] Izdebski R. Fiett J. Urbanowicz P. Baraniak A. Derde L. P. G. Bonten M. J. M. (2015). Phylogenetic lineages, clones and β-lactamases in an international collection of *Klebsiella oxytoca* isolates non-susceptible to expanded-spectrum cephalosporins. *J. Antimicrob. Chemother.* 2015:dkv273. 10.1093/jac/dkv273 26318191

[B17] Joainig M. M. Gorkiewicz G. Leitner E. Weberhofer P. Zollner-Schwetz I. Lippe I. (2010). Cytotoxic Effects of *Klebsiella oxytoca* Strains Isolated from Patients with Antibiotic-Associated Hemorrhagic Colitis or Other Diseases Caused by Infections and from Healthy Subjects. *J. Clin. Microbiol.* 48 817–824. 10.1128/jcm.01741-09 20053860 PMC2832427

[B18] Kumar S. Stecher G. Li M. Knyaz C. Tamura K. (2018). MEGA X: Molecular Evolutionary Genetics Analysis across Computing Platforms. *Mol. Biol. Evolut.* 35 1547–1549. 10.1093/molbev/msy096 29722887 PMC5967553

[B19] Kurtz S. Phillippy A. Delcher A. L. Smoot M. Shumway M. Antonescu C. (2004). Versatile and open software for comparing large genomes. *Genome Biol.* 5:R12. 14759262 10.1186/gb-2004-5-2-r12 PMC395750

[B20] Li J. Oh J. Kienesberger S. Kim N. Y. Clarke D. J. Zechner E. L. (2020). Making and Breaking Leupeptin Protease Inhibitors in Pathogenic Gammaproteobacteria. *Angew Chem. Int. Ed.* 59 17872–17880. 10.1002/anie.202005506 32609431

[B21] Lowe C. Willey B. O’Shaughnessy A. Lee W. Lum M. Pike K. (2012). Outbreak of Extended-Spectrum β-Lactamase–producing *Klebsiella oxytoca* Infections Associated with Contaminated Handwashing Sinks1. *Emerg. Infect. Dis.* 18 1242–1247. 10.3201/eid1808.111268 22841005 PMC3414015

[B22] Merla C. Rodrigues C. Passet V. Corbella M. Thorpe H. A. Kallonen T. V. S. (2019). Description of *Klebsiella spallanzanii* sp. nov. and of *Klebsiella pasteurii* sp. nov. *Front. Microbiol.* 10:2360. 10.3389/fmicb.2019.02360 31708881 PMC6824210

[B23] Moradigaravand D. Boinett C. J. Martin V. Peacock S. J. Parkhill J. (2016). Recent independent emergence of multiple multidrug-resistant *Serratia marcescens* clones within the United Kingdom and Ireland. *Genome Res.* 26 1101–1109. 10.1101/gr.205245.116 27432456 PMC4971767

[B24] Moradigaravand D. Martin V. Peacock S. J. Parkhill J. (2017). Population Structure of Multidrug-Resistant *Klebsiella oxytoca* within Hospitals across the United Kingdom and Ireland Identifies Sharing of Virulence and Resistance Genes with *K. pneumoniae*. *Genome Biol. Evolut.* 9 574–584. 10.1093/gbe/evx019 28177070 PMC5381567

[B25] Oliveira R. A. Ng K. M. Correia M. B. Cabral V. Shi H. Sonnenburg J. L. (2020). *Klebsiella michiganensis* transmission enhances resistance to Enterobacteriaceae gut invasion by nutrition competition. *Nat. Microbiol.* 5 630–641. 10.1038/s41564-019-0658-4 31959968

[B26] Passet V. Brisse S. (2018). Description of *Klebsiella grimontii s*p. nov. *Int. J. Systemat. Evolut. Microbiol.* 68 377–381. 10.1099/ijsem.0.002517 29205126

[B27] Paveglio S. Ledala N. Rezaul K. Lin Q. Zhou Y. Provatas A. A. (2020). Cytotoxin-producing *Klebsiella oxytoca* in the preterm gut and its association with necrotizing enterocolitis. *Emerg. Microbes Infect.* 9 1321–1329. 10.1080/22221751.2020.1773743 32525754 PMC7473113

[B28] Pritchard L. Glover R. H. Humphris S. Elphinstone J. G. Toth I. K. (2016). Genomics and taxonomy in diagnostics for food security: soft-rotting enterobacterial plant pathogens. *Anal. Methods* 8 12–24. 10.1039/c5ay02550h

[B29] Rønning T. G. Aas C. G. Støen R. Bergh K. Afset J. E. Holte M. S. (2019). Investigation of an outbreak caused by antibiotic−susceptible *Klebsiella oxytoca* in a neonatal intensive care unit in Norway. *Acta Paediatr.* 108 76–82. 10.1111/apa.14584 30238492

[B30] Saha R. Farrance C. E. Verghese B. Hong S. Donofrio R. S. (2013). *Klebsiella michiganensis* sp. nov., A New Bacterium Isolated from a Tooth Brush Holder. *Curr. Microbiol.* 66 72–78. 10.1007/s00284-012-0245-x 23053492

[B31] Segata N. Börnigen D. Morgan X. C. Huttenhower C. (2013). PhyloPhlAn is a new method for improved phylogenetic and taxonomic placement of microbes. *Nat. Commun.* 4:2304. 23942190 10.1038/ncomms3304 PMC3760377

[B32] Singh L. Cariappa M. P. Kaur M. (2016). *Klebsiella oxytoca*: An emerging pathogen? *Med. J. Armed Forces India* 72 S59–S61. 28050072 10.1016/j.mjafi.2016.05.002 PMC5192185

[B33] Unterhauser K. Pöltl L. Schneditz G. Kienesberger S. Glabonjat R. A. Kitsera M. (2019). *Klebsiella oxytoca* enterotoxins tilimycin and tilivalline have distinct host DNA-damaging and microtubule-stabilizing activities. *Proc. Natl. Acad. Sci. U S A.* 116 3774–3783. 10.1073/pnas.1819154116 30808763 PMC6397511

[B34] Validi M. Soltan Dallal M. M. Douraghi M. Fallah Mehrabadi J. Rahimi Foroushani A. (2016). Identification of *Klebsiella pneumoniae* Carbapenemase-producing *Klebsiella oxytoca* in Clinical Isolates in Tehran Hospitals, Iran by Chromogenic Medium and Molecular Methods. *Osong Public Health Res. Perspect.* 7 301–306. 27812488 10.1016/j.phrp.2016.08.006 PMC5079191

[B35] Vasaikar S. Obi L. Morobe I. Bisi-Johnson M. (2017). Molecular Characteristics and Antibiotic Resistance Profiles of *Klebsiella* Isolates in Mthatha, Eastern Cape Province, South Africa. *Int. J. Microbiol.* 2017 1–7. 10.1155/2017/8486742 28250772 PMC5303861

