# Corrigendum: Variation in accessory genes within the *Klebsiella oxytoca* species complex delineates monophyletic members and simplifies coherent genotyping

**DOI:** 10.3389/fmicb.2023.1155851

**Published:** 2023-03-07

**Authors:** Amar Cosic, Eva Leitner, Christian Petternel, Herbert Galler, Franz F. Reinthaler, Kathrin A. Herzog-Obereder, Elisabeth Tatscher, Sandra Raffl, Gebhard Feierl, Christoph Högenauer, Ellen L. Zechner, Sabine Kienesberger

**Affiliations:** ^1^Institute of Molecular Biosciences, University of Graz, Graz, Austria; ^2^BioTechMed-Graz, Graz, Austria; ^3^Diagnostic and Research Institute of Hygiene, Microbiology and Environmental Medicine, Medical University of Graz, Graz, Austria; ^4^Division of Gastroenterology and Hepatology, Department of Internal Medicine, Medical University of Graz, Graz, Austria; ^5^Field of Excellence BioHealth, University of Graz, Graz, Austria

**Keywords:** bacterial phylogeny, *Klebsiella oxytoca* species complex, taxonomic classification, necrotizing enterocolitis, bacterial cytotoxicity, intestinal disease

In the published article, there was an error in Table 1 as published. An incorrect oligonucleotide sequence was provided for primer #10 core_r. The corrected Table 1 and its caption appear below.

**Table 1:** Oligonucleotides used in PCR-screening and sequencing.

**#**	**Primer name**	**Sequence 5^′^-3^′^**	**Binding Site (nt position) in**	**Reference sequence**	**Fragment size**	**Reference**
1	npsB_f	ctcgacgttttatctctgctg	20117..20137	AHC-6^*^	231 bp	This study
2	npsB_r	ttcctgaagtatctgccctgc	20327..20347			This study
3	OXY1-A	gtggcgtaaaaccgccctg	5001082..5001100	CAV1374	425 bp	Fevre et al., 2005
4	OXY1-B	gtccgccaaggtagctaatc	5001487...5001506			Fevre et al., 2005
5	OXY2-A	aaggctggagattaacgcag	4572112..4572131	NCTC13727	155 bp	Fevre et al., 2005
6	OXY2-B	gcccgccaaggtagccgatg	4572247..4572266			Fevre et al., 2005
7	OXY-E	ggttt**T**ggtaactgtgacggg	5304405..5304937	NCTC13727	1,098 bp	Fevre et al., 2005
8	OXY-G	cagagt**G**cagagtgttgcag	5304405..5304937			Fevre et al., 2005
9	core_f	gagatcccaagttctttagcaatgg	227821..227845	NCTC13727	175 bp	This study
10	core_r	agcacgttttccaggcgctgg	227976..227995			This study
11	leupA_f	atgaagatagcgattcacaac	315403..315425	CAV1374	1,235 bp	Li et al., 2020
12	leupB_r	gcgtggtcttttagctgttc	316620..316639			Li et al., 2020
13	orfA_f	gcagtgatttaaatactcttgcgg	53092..53115	JKo3	2,525 bp	This study
14	orfC_r	ccgatacctccagtaatgcgc	55617..55637			This study
15	B2_v2_1	cggcttacgcacaaagaagcc	74467..74487	ARO112, cont20	591 bp	this Study
16	B2_v2_2	atgtttcttgaagaacgtagg	75037..75075			This study
17	gapA_fwd	gttttcccagtcacgacgttgtatgaagtatgactccactcacgg	4012172..4012193	CAV1374	680 bp	Herzog et al., 2014
18	gapA_rev	ttgtgagcggataacaatttcaacgcctttcattgcgccttcggaa	4012827..4012851			Herzog et al., 2014
19	infB_fwd	gttttcccagtcacgacgttgtactctctgctggactacattcg	1034476..1034496	CAV1374	463 bp	Herzog et al., 2014
20	infB_rev	ttgtgagcggataacaatttccgctttcagctccagaacttc	1024918..1034938			Herzog et al., 2014
21	mdh_fwd	gttttcccagtcacgacgttgtacccaactgccttcaggttcag	964459..964479	CAV1374	704 bp	Herzog et al., 2014
22	mdh_rev	ttgtgagcggataacaatttc**C**cttc**C**acgtaggcgcattcc	965141..965162			Herzog et al., 2014
23	pgi_fwd	gttttcccagtcacgacgttgtagagaaaaacctgccggtgctgctg	21866..21889	CAV1374	701 bp	Herzog et al., 2014
24	pgi_rev	ttgtgagcggataacaatttccggttaatcag**G**ccgttagtggagc	21189..21213			Herzog et al., 2014
25	phoE_fwd	gttttcccagtcacgacgttgtaacct**GG**cgcaa**C**accga**T**ttcttc	5304914..5304937	CAV1374	533 bp	Herzog et al., 2014
26	phoE_rev	ttgtgagcggataacaatttcttcagctggttgattttgtaatccac	5304405..5304430			Herzog et al., 2014
27	rpoB_fwd	gttttcccagtcacgacgttgtaggcgaaatggcggaaaacca	111644..111663	CAV1374	1,076 bp	Herzog et al., 2014
28	rpoB_rev	ttgtgagcggataacaatttcgagtcttcgaagttgtaacc	110588..110607			Herzog et al., 2014
29	tonB_fwd	gttttcccagtcacgacgttgtactctatacttcggtacatcaggtt	2890556..2890579	CAV1374	589 bp	Herzog et al., 2014
30	tonB_rev_2	ttgtgagcggataacaatttcgtttacccggttcatagcgcc	2891124..2891144			This study
31	tonB_rev	ttgtgagcggataacaatttccctgtttggcggccagcacctggt	2686207..2686230	NCTC13727^**^	740 bp	Herzog et al., 2014
32	MLST_seq_fwd	gttttcccagtcacgacgttgta	n/a	n/a	n/a	Herzog et al., 2014
33	MLST_seq_rev	ttgtgagcggataacaatttc	n/a	n/a	n/a	Herzog et al., 2014

^*^Tilimycin/tilivalline biosynthesis gene cluster (accession number: HG425356.1). ^**^Only first 4 nucleotides on the 3'end of the primer bind in CAV1374 in the first reading frame after *tonB* (in the fructosamine kinase family protein), therefore primer binding site is given for NCTC13727. underlined: binding site for MLST_seq primers; bold: primer binding site ambiguity in given reference seq.

The authors apologize for this error and state that this does not change the scientific conclusions of the article in any way. The original article has been updated.
